# Long-term outcomes after single-stage augmentation mastopexy: A ten-year series and risk stratification model

**DOI:** 10.1016/j.jpra.2026.02.026

**Published:** 2026-02-28

**Authors:** James Lucocq, Taimur Shoaib

**Affiliations:** a Edinburgh Breast Unit, Western General Hospital, Edinburgh, UK; b La Belle Forme Clinic, Glasgow, UK

**Keywords:** Augmentation, Mastopexy, Complications, Risk factors

## Abstract

**Introduction:**

The existing literature on single-stage augmentation mastopexy is largely limited to short follow-up (<1 year), single surgeon series or outcomes in highly specific subgroups. The study aims to report long-term contemporary outcomes for single-stage augmentation mastopexy and to propose a risk stratification tool.

**Methods:**

Ten-year outcomes of consecutive cases of single-stage augmentation mastopexy between 2014–2024 were reported from a single center. Multivariate logistic regression identified risk factors for complication and re-operation and a risk-stratification tool was proposed.

**Results:**

289 cases of single-stage augmentation mastopexy (Wise, 40.5% [117/289]; vertical with lateral extension, 26.3% [76/289]; vertical, 24.6% [71/289]) were performed including 45 patients (15.6%) with a history of previous augmentation or mastopexy. At a median follow-up of 46 months, implant- and tissue-related related complications occurred in 6.6% (19/289) and 26.0% (75/289) of cases, respectively. Re-operation occurred in 19.4% (56/289), of which 21.6% (12/56) were performed under local anaesthetic. Re-operation rates under general anaesthetic were 10.6%, 14.6% and 18.4% at 1, 3 and 5 years, respectively. The most common re-operation was revision mastopexy (11/289, 3.8%), followed by nipple areolar complex revision (9/289, 3.1%). Previous augmentation or mastopexy (OR 3.58; 95% CI,1.27–10.07), high-projection implants (OR 1.84; 95% CI,1.02–3.32) and smoking (OR 2.46; 95% CI,1.13–5.33) were associated with complication and/or re-operation. Landmark analysis identified implant-related complications in 4.8% (5/104) beyond 3 years and 2.0% (1/50) after 5 years, while tissue-related complications occurred in 1.0% (1/104) after 3 years and were absent beyond 5 years.

**Conclusions:**

Single-stage augmentation mastopexy can be performed safely in well-selected patients with promising long-term complication and re-operation rates.

## Introduction

Single-stage augmentation mastopexy is a complex surgical procedure that combines volume enhancement and ptosis correction simultaneously, while attempting to reduce the need for a subsequent operation.^1^^,^^2^ The procedure is indicated for hypoplastic and ptotic breasts, with excess of the skin envelope. It is inherently challenging due to the opposing forces involved and the conflicting goals of increasing breast volume and height, while simultaneously reducing the skin envelope. The risks of a single-stage are compounded by the individual risks of breast augmentation (e.g. capsular contracture) and mastopexy (e.g. recurrent ptosis, wound healing issues), and consequently it has one of the highest litigation rates amongst plastic surgical procedures.^2^, ^3^, ^4^, ^5^

Single-stage augmentation mastopexy offers a single recovery period, quicker aesthetic results, and lower cost, compared with two-stage approach.^6^ Contrarily, two-stage surgery, typically mastopexy followed by augmentation after 3–6 months, subjects the patient to a guaranteed second procedure and higher overall cost. The potential advantages of a single-stage approach may be offset by higher overall morbidity and a less predictable outcome; however, this is largely speculative and has not been demonstrated definitively.^2^^,^^7^, ^8^, ^9^

The existing literature on single-stage augmentation mastopexy is largely limited to short follow-up (<1 year), single surgeon series or outcomes in highly specific subgroups (e.g. post massive weight loss).^2^^,^^10^ Furthermore, there remains a need to better identify patients, based on patient- and procedure-specific factors, in whom a single-stage approach can be undertaken with acceptable morbidity. The primary aims of this study were to report long-term contemporary outcomes for single-stage augmentation mastopexy and to propose a risk stratification tool to inform patient consent and aid decision-making.^1^^,^^11^

## Method

All cases of single-stage augmentation mastopexy performed in our center, including five surgeons, between 2014 and 2024 were reported retrospectively. Primary and secondary augmentation mastopexy were included. All elective procedures within the unit were limited to patients with American Society of Anaesthesiologists (ASA) physical status scores of 1 or 2, in accordance with our institution’s current day-case pathway and with previous risk management eligibility criteria. All patients were reviewed in a consultation prior to surgery and were noted to have ptotic hypoplastic breasts. Implant size, projection and position (e.g. subglandular versus submuscular) were determined pre-operatively and individualized by accounting for the patients’ anatomical measurements, desire for upper pole fullness and physical activity. All patients were advised to stop smoking at the time of consultation, if applicable, for at least 6–8 weeks prior to surgery.

All cases of single-stage augmentation mastopexy were managed as day-cases within the unit. Routine follow-up occurred approximately at 6 weeks, 6 months and 1 year. Thereafter patients were given an open appointment to present if required and were reviewed again if clinically indicated or at the patient’s request. Our institutional policy offers free revisional surgery, under either local or general anaesthetic, when the operating surgeon determined that further improvement was achievable. For the purposes of this study, follow-up duration was defined as the time from the index procedure to the most recent review of the electronic patient record at the time of data extraction.

In our unit, a single-stage approach was routine practice for patients with ptotic hypoplastic breasts, and the two-stage approach was reserved for cases considered too high risk or in line with patient preference. As a default, no patients were routinely listed for a single-stage augmentation mastopexy if they had any medical conditions which could impact the vascularity or tissue healing (e.g., clotting or bleeding disorders). Furthermore, patients with marked loss of dermal elasticity or severe envelope laxity were counseled regarding the increased risk of recurrent ptosis and waterfall deformity following a single-stage approach, as described in previous series, and a staged approach was discussed in an attempt to reduce complications.^12^, ^13^, ^14^ For context, only fourteen patients underwent a planned two-stage augmentation mastopexy within the unit. Review of clinical records indicates that reasoning for staging was largely multifactorial due to poor skin quality (*n* = 8), significant smoking history (*n* = 5), patient preference (*n* = 5) and previous surgery (*n* = 3).

### Data analysis

Pre-operative patient variables were collected including previous breast procedures, smoking status, American Society of Anaesthesiologists (ASA) score and anatomical data (e.g., Sternal Notch to Nipple [SN-N] distance). Either cigarette smoking or vaping at the time of consultation were considered. Operative variables included implant position, pedicle and skin incision. Implant variables were also collected including implant type (e.g., round vs. anatomical), brand, projection and size.

Each case was followed up until the present day for complications which were categorized as implant-related or tissue-related and graded according to the Clavien-Dindo classification.^15^ Complications requiring re-operation were reported according to the need for general (GA) or local anaesthetic (LA). The 1-, 3- and 5-year complication and re-operation rates were reported using Kaplan-Meier curves. Landmark analyses were performed at 3 years and 5 years to evaluate the conditional risk of late events from each time point. For each outcome, patients who were event-free and under follow-up at the landmark timepoint were included.

### Surgical technique

Although five surgeons performed the operations during the time-period, the senior author (TS) performed most of the operations (*n* = 195; 67.5%), and his surgical technique is described in detail in the supplementary methods. The remaining cases were performed by four surgeons who held part-time appointments within the unit, were appointed later in the study period, and concurrently practiced at other centers. Nevertheless, a number of similarities were present amongst the included surgeons: 1) all surgeons defaulted to a superior pedicle where possible; 2) superior-medial pedicles were selected if a superior pedicle was considered excessively long; 3) an approximate 2 cm threshold of upper pole thickness was used to guide implant pocket position; and 4) implant dimensions were guided by the breast width and the desired breast height. A standardized peri-operative protocol was used in the unit including: 1) prophylactic IV antibiotics given at induction (Cefuroxime 1.5 g); 2) tranexamic acid (1 g IV); 3) antiseptic irrigation of the implant pocket (Gentamicin 40 mg in 10 mL 0.5% bupivacaine), and 4) a post-surgical bra was worn for 6 weeks post-operatively. Both round and anatomical implants were used across the study period and in all cases the implants were either smooth or microtextured. A combination of the Wise, vertical and periareolar mastopexies were used according to the skin-excess and degree of ptosis. Both Wise and vertical have been regarded as safe in terms of nipple vascularity.^16^, ^17^, ^18^, ^19^

### Risk factors for complications

Risk factors associated with implant-related complications, tissue-related complications and re-operation were identified using multivariate logistic regression. Clinically relevant variables were included in the regression as follows: 1) Implant volume due to its possible relationship with recurrent ptosis and tissue related complication. This was modeled as a continuous variable (per 50 cc increase), rather than categorized using a fixed threshold since there is no universally accepted threshold.^14^^,^^20^^,^^21^ 2) Implant projection was included in view of the potential for implant dimensions to influence implant malposition and tissue-related complications.^22^^,^^23^ 3) Smoking status given its association with poor tissue perfusion and delayed wound healing.^9^^,^^21^^,^^24^ 4) Previous augmentation or mastopexy due to the risk of biofilm and tissue compromise.^25^

### Risk stratification tool

A simple risk stratification tool for single-stage augmentation mastopexy is proposed, incorporating variables independently associated with implant-related complications, tissue-related complications, or re-operation on multivariable logistic regression, alongside established clinical factors already used in practice (e.g., conditions affecting tissue vascularity and poor skin quality). The purpose of this tool is to support informed consent and shared decision-making by identifying patients in whom a single-stage approach may be undertaken with acceptable morbidity. All statistical analysis was conducted in RStudio Version 2024.04.2 + 764. A *p*-value < 0.05 was considered statistically significant and two tailed tests were used.

## Results

Over a 10-year period, between 2014 and 2024, single-stage bilateral augmentation mastopexy was performed in 289 patients in our center by five surgeons. Background characteristics are reported in Table 1. The median age was 34.5 years (Q1-3, 28-42) and all patients had either an ASA of 1 or 2. Forty-eight patients (16.6%) had previous breast surgery, the most common of which was a previous breast augmentation (*n* = 33, 11.4%). Eleven patients (3.8%) had undergone a previous augmentation mastopexy. The median SN-N distance was 24.5 cm and 23 patients (8.0%) had an upper pole fullness of less than 2 cm.

**Table 1:** Background characteristics of the primary cohort.

Variable		Number, *n* = 289 (%)
Median age		34.5, 28–42
Previous breast surgery	All procedures	48 (16.6)
	*Augmentation*	33 (11.4)
	*Augmentation mastopexy*	12 (4.2)
	*Fat transfer*	2 (0.7)
	*Wide local excision*	1 (0.3)
Reported cause for change in breast shape (if applicable)	Weight loss	68 (23.5)
	Pregnancy changes	42 (14.5)
	Breast feeding	17 (5.9)
Breast width, Median, Q1-3		13 cm, 12–14 cm
Breast height Median, Q1-3		9 cm
SN-N distance, Median,		24.5 cm, 23–26 cm
SN-N asymmetry ≥1 cm		68 (23.5)
Upper pole thickness <2 cm		23 (8.0)
Pseudoptosis		3 (1.0)
ASA	1	253 (87.5)
	2	36 (12.5)
Smokers		31 (10.7)

Most of the implants were subglandular (*n* = 204, 70.6%), with a superior pedicle (*n* = 234, 81.0%) and the most common skin excision pattern was an inverted-T, Wise-type pattern (*n* = 117, 40.5%), followed by vertical/lateral extensions/J-L shaped scar (*n* = 76, 26.3%) and vertical scar (*n* = 71, 24.6%). Operative and implant details are reported in Table 2.

**Table 2:** Operative details of the primary cohort.

Operative detail		Number, *n* = 289 (%)
Position	Subglandular	204 (70.6)
	Dual Plane	42 (14.5)
	Subfascial	17 (5.9)
	Submuscular	15 (5.2)
Pedicle	Superior	234 (81.0)
	Superio-medial	29 (10.0)
	Central	19 (6.6)
	Lateral and medial	1 (0.3)
Skin incision	Wise pattern	117 (40.5)
	Vertical and lateral extension	76 (26.3)
	Vertical	71 (24.6)
	Peri-areolar	19 (6.6)
	Other	6 (2.1)
Implant type	PERLE	152 (52.6)
	Allergan	47 (16.3)
	NAGOR	40 (13.8)
	Motiva	35 (12.1)
	Other	15 (5.2)
Implant shape	Round	236 (81.7)
	Anatomical	49 (16.9)
High projection implant		111 (38.4)
Implant size, median, Q1-3		315, 280–340 cc
Implant size	≤250 cc	32 (11.1)
	251–350 cc	198 (68.5)
	351–450 cc	48 (16.6)
	>450 cc	5 (1.7)
Drain		6 (2.1)

### Complications

The median follow-up for complications was 46 months (Q1-3, 20 – 90) and overall, 30.4% (88 patients) suffered complications (Table 3). Eighty patients (90.9%) had unilateral complications, and 8 patients (9.1%) had bilateral complications; thus the complication rate per breast was 16.6% (96/578).

**Table 3:** Complication and Re-operations during follow-up of the primary cohort.

Outcome		Number, *n* = 289 (%)
Complications (all)	Tissue related	75 (26.0)
	*Minor wound breakdown or dehiscence*	24 (8.3)
	*Hypertrophic scarring*	15 (5.2)
	*NAC/Breast asymmetry*	11 (4.2)
	*Recurrent ptosis or bottoming out*	10 (3.5)
	*Hematoma*	9 (3.1)
	*Waterfall deformity or excess skin*	2 (0.7)
	*Fat necrosis*	2 (0.7)
	*Double bubble*	1 (0.3)
	*Partial nipple necrosis*	1 (0.3)
	*Full nipple necrosis*	0 (0.0)
	Implant related	19 (6.6)
	*Capsular Contracture*	9 (3.1)
	*Implant malposition ± immobility*	6 (2.1)
	*Implant infection without hematoma*	4 (1.4)
	*Implant exposure/seroma/rippling/failure*	0 (0.0)
Re-operation	All	56 (19.4)
	General Anaesthetic	44 (15.2)
	Local Anaesthetic	12 (4.2)
Re-operation (Clavien-Dindo ≥3)	Revision mastopexy ± capsulectomy ± exchange of implant	11 (3.8)
	Evacuation ± Hemostasis	9 (3.1)
	NAC revision	9 (3.1)
	Repositioning of implant	6 (2.1
	Skin envelope/Scar revision	6 (2.1)
	Capsulectomy ± Exchange/Removal of implant	6 (2.1)
	Washout ± debridement	6 (2.1)
	Exchange/Removal of implant	3 (1.0)

Tissue-related and implant related complications occurred in 26.0% (n=75) and 6.6% (n=19) of patients, respectively. The most common tissue-related complications were minor wound breakdown or dehiscence in 24 patients (8.3%), hypertrophic scarring in 15 patients (5.2%) and nipple areolar asymmetry in 11 patients (4.2%). The most common implant-related complication was capsular contracture in 9 patients (3.1%) followed by implant malposition in 6 patients (2.1%).

**Figure 1:**
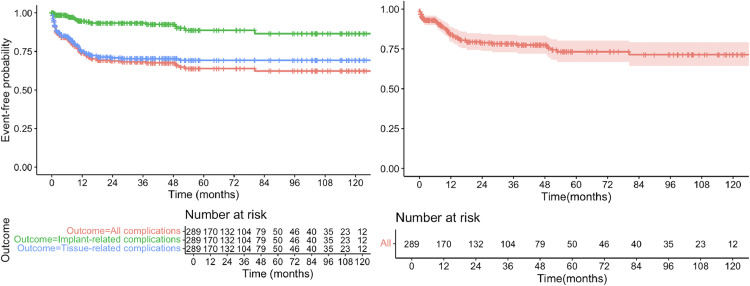
The long-term risk of complications (A) and re-operation (B).

Rates of tissue and implant-related complications over time are illustrated in Figure 1a. The 1-, 3- and 5-year rate of implant-related complication was 4.4%, 6.6% and 11.3%; compared with 25.0%, 29.9% and 30.7% for tissue-related complications. The landmark analysis identified 5 cases of implant complication and one tissue-related complication (1/104, 1.0%) from 3-years onwards in those who are complication free by 3 years (5/104, *n* = 4.8%). One implant related complication was identified (1/50, 2%), but no tissue complications were recorded after 5 years in those who are complication-free at 5-years. (Supplementary Figure 1).

### Re-operation

A total of 56 patients (19.4%) suffered a complication requiring re-operation (Clavien-Dindo≥3), of which 44 (15.2%) were managed under GA and the remaining under LA (4.2%; *n* = 12). The 1-, 3- and 5-year re-operation rate was 14.6%, 19.4% and 23.2%, respectively (Figure 1b); whereas the 1-, 3- and 5-year re-operation rate under GA was significantly lower (10.6%, 14.6% and 18.4%, respectively).

Of those undergoing a re-operation, 67.9% (38 patients) were for a tissue-related complication; 19.6% (11 patients) for an implant complication and 12.5% (7 patients) for both an implant and tissue-related complication.

The most common re-operation was a revision mastopexy ± capsulectomy ± exchange of implant (*n* = 11, 3.8%) followed by nipple revision (*n* = 9, 3.1%) and evacuation of hematoma (*n* = 9, 3.1%) (Table 3). Washout ± tissue debridement, capsulectomy ± implant exchange/removal and scar revision were each required in six (2.1%) patients. Of the 12 procedures performed under LA, nine were for nipple areola complex (NAC) revisions and three were for scar revisions.

### Risk factors for complications

Multivariate logistic regression was performed using the following variables: smoking status, high versus moderate profile implants, implant size (per 50 cc) and previous augmentation or mastopexy (Table 4). On multivariable logistic regression, previous augmentation or mastopexy (OR 3.58, 95% CI 1.27–10.07; *p* = 0.016) and high-projection implants (OR 4.88, 95% CI 1.63–14.60; *p* = 0.005) were independently associated with implant-related complications. Smoking (OR 2.46, 95% CI 1.13–5.33; *p* = 0.023) and high-projection implants (OR 1.84, 95% CI 1.02–3.32; *p* = 0.044) were independently associated with increased odds of tissue-related complication. Previous augmentation or mastopexy was independently associated with re-operation (OR 2.08, 95% CI 1.02–4.24; *p* = 0.045).

**Table 4:** Multivariate logistic regression investigating variables associated with implant complications, tissue complications and re-operation.

	Predictor	Odds ratio (OR)	95% CI	*z* value	*p* value
Implant complications	Smoker	1.64	0.44–6.10	0.73	0.466
	Previous augmentation or mastopexy	**3.58**	**1.27–10.07**	**2.42**	**0.016**
	High projection implant	**4.88**	**1.63–14.60**	**2.83**	**0.005**
	Implant size (per 50 cc)	0.99	0.98–1.00	−1.43	0.153
Tissue complications	Smoker	**2.46**	**1.13–5.33**	**2.27**	**0.023**
	Previous augmentation or mastopexy	1.32	0.66–2.65	0.78	0.436
	High projection implant	**1.84**	**1.02–3.32**	**2.02**	**0.044**
	Implant size (per 50 cc)	1.00	0.99–1.01	0.44	0.660
Re-operation	Smoker	1.70	0.73–3.95	1.22	0.221
	Previous augmentation or mastopexy	**2.08**	**1.02–4.24**	**2.01**	**0.045**
	High projection implant	1.57	0.82–2.99	1.35	0.178
	Implant size (per 50 cc)	1.00	0.99–1.01	−0.16	0.873

The bold values are statistically significant (*p* < 0.05).

**Figure 2:**
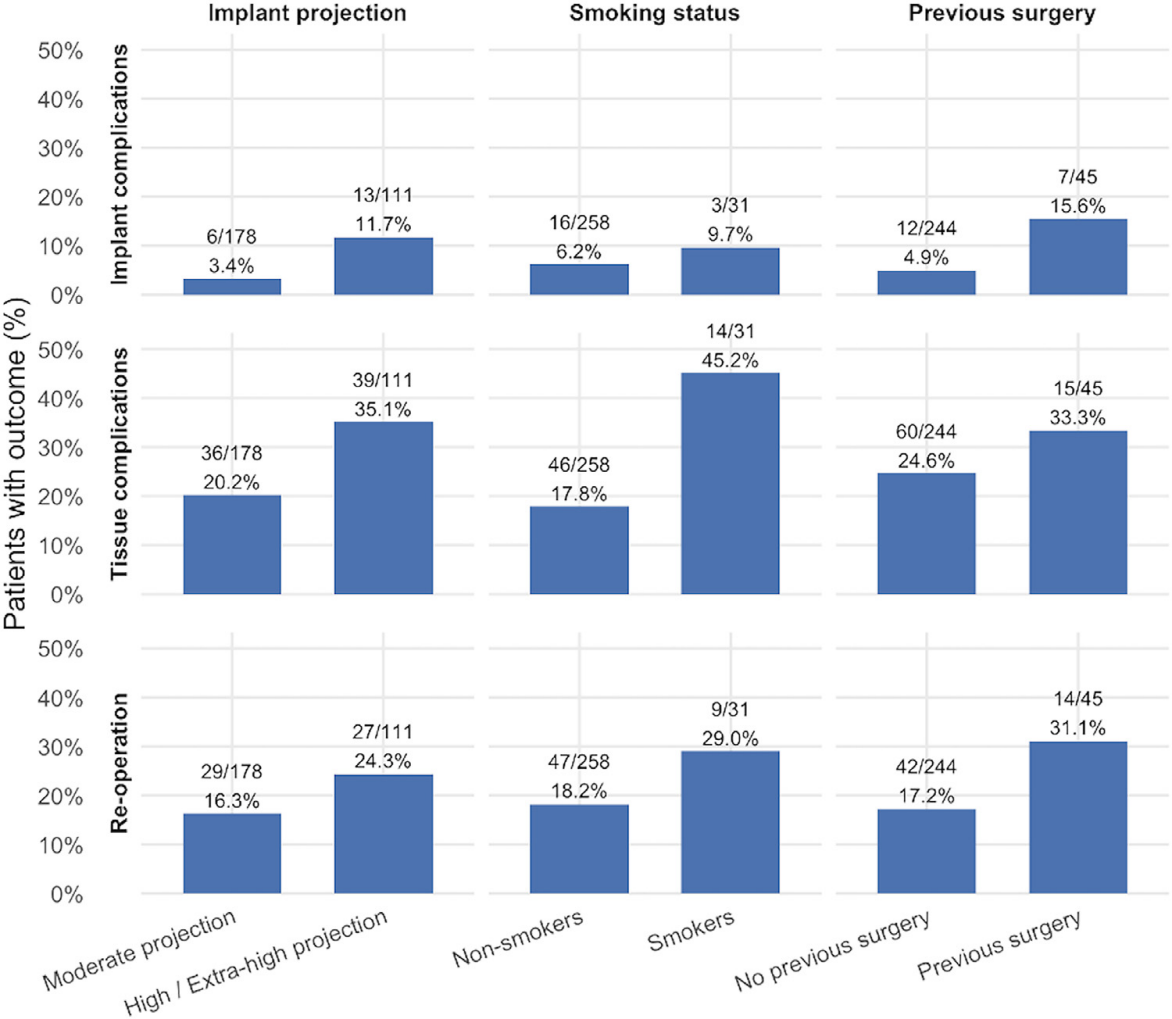
The risk of complication and re-operation by smoking status, implant projection and previous breast surgery.

**Figure 3:**
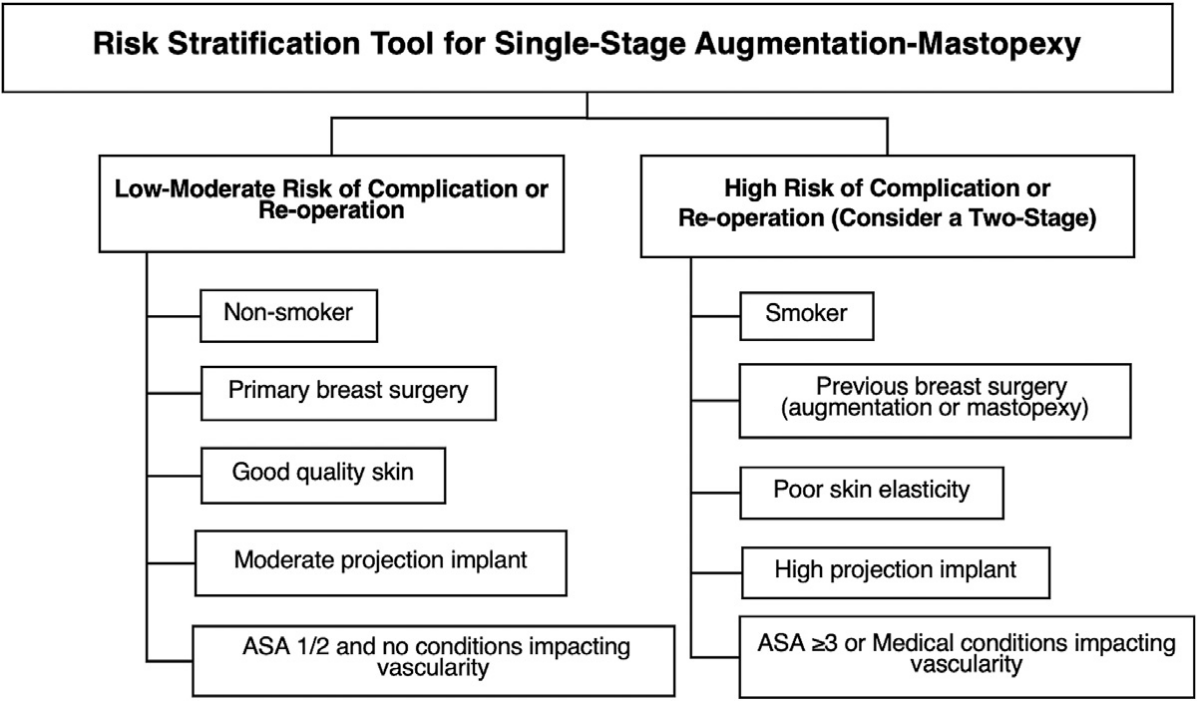
Risk stratification tool for single-stage augmentation mastopexy.

The risks of complication and re-operation by smoking status, previous augmentation/mastopexy and implant projection are reported in Figure 2. The proposed risk stratification tool is illustrated in Figure 3.

## Discussion

Single-stage augmentation mastopexy is a technically demanding procedure due to the conflicting goals of increasing breast volume while simultaneously reducing the skin envelope. Concerns have historically been raised regarding the increased perceived risk of significant complications, such as NAC necrosis, implant extrusion, and tissue devascularization, following this procedure.^26^^,^^27^ Although both complications and re-operations were not uncommon during long-term follow-up in this large series of 289 patients, serious complications were extremely infrequent across long-term follow-up. Only one case of partial nipple necrosis was observed, there were no cases of implant extrusion and the implant infection rate was low (*n* = 4). In fact, many of the re-operations could be managed under a local anaesthetic (21.6% of operations), a detail often not reported in the relevant literature. Only 15.2% required procedures under general anaesthetic and this equated to a 18.4% risk at 5 years. Overall, despite the longer follow-up presented here, these outcomes are broadly similar to those presented in a meta-analysis of 19 studies comprising of 5837 patients.^2^^,^^21^^,^^28^

The study has reported long-term outcomes and trends in complications rates following single-stage augmentation-mastopexy over 10 years. Interestingly, the re-operation rate under general anaesthetic rose by almost 8% between 1- and 5-years (10.6% vs. 18.4%) which indicates that many of the shorter-term studies reporting complications have most likely underestimated the complication rate. Although the rate of implant complication was very low overall (6.6%), most of the later complications (beyond year 3) were implant-related and mostly reflect capsular contracture or implant malposition. Nevertheless, beyond 5 years, the Kaplan-Meier curves had plateaued reflecting stable results and low long-term revision rates.

The fundamental question is whether the morbidity of the single-stage approach is a safe alternative to the guaranteed morbidity of a planned second-stage procedure and its additional costs. Over the past 2 decades, the high re-operation rates of single-stage have often been considered in excess of the summative rates of consecutive procedures.^8^^,^^9^^,^^18^^,^^21^^,^^29^ On the other hand, other authors such as Swanson et al. suggest that a single stage may in fact lower the cumulative complication rate, particularly when a vertical mastopexy approach is used, even in cases of challenging morphology.^19^^,^^30^ The substantial heterogeneity in surgical technique (e.g., implant selection, plane, skin excision pattern, pedicle choice) between published series, helps explain the variability in reported outcomes and difficulty in forming a direct comparison.^19^ Without the appropriate methodology design to address this clinical question, conclusions surrounding their relative morbidity remain speculative.^27^^,^^31^ Nevertheless, these uncertainties do not preclude the identification of lower-risk patients in whom a single-stage approach can be performed with acceptably low morbidity, allowing them to benefit from reduced cost and a single recovery period.

It is well established that tissue-related complication rates are significantly higher than implant-related complications following augmentation-mastopexy.^2^^,^^7^^,^^9^^,^^32^ Although nipple necrosis was rare (*n* = 1), less severe complications such as minor wound breakdown (8.3%) and poor scarring (5.2%) were common. Smoking is a well-reported risk-factor for these complications, including in this series, explained by compromised microvascular perfusion and delayed wound healing.^9^^,^^21^^,^^24^ In our practice both smokers and recent ex-smokers were advised to stop smoking for at least 6–8 weeks pre-operatively, yet were still predisposed to these complications and nearly a 30% re-operation rate. Patients with poor skin quality, often secondary to massive weight loss, also represent a high-risk group for tissue-related complications. This is often indicated by the presence of stretch marks and/or a significant skin stretch measurements on examination.^13^^,^^33^ Despite aggressive skin excision, recurrent ptosis and lower pole stretch are common often warranting secondary mastopexy.^7^^,^^12^, ^13^, ^14^ These high-risk patients should be counseled regarding their substantially increased risk of aesthetic complications, with the relative merits of single- versus two-stage surgery discussed as part of shared decision-making.

Large breast volume and larger implant have been implicated as risk factors in augmentation mastopexy.^7^^,^^14^^,^^20^^,^^21^ The combined weight of the breast and implant hypothetically predispose to lower pole stretch, recurrent ptosis and possibly implant malposition.^7^^,^^14^^,^^20^^,^^21^ In our analysis, higher implant volume was not a risk factor but was likely confounded by high implant projection which was associated with tissue complications in 35%. In theory both high projection and implant volume could exacerbate the conflicting forces of skin-tightening and volume augmentation risking wound breakdown, dehiscence, NAC displacement, and even recurrent ptosis. Therefore, caution is advised in patients desiring more upper-pole fullness necessitating a high-profile implant with a wider base, particularly where there is concern of the skin elasticity. A more conservative implant selection may mitigate against these complications in single-stage cases with borderline tissue quality. Whether staging allows the surgeon to reassess the breast envelope and revise vertical limb markings based on tissue adaptation is possible but unclear.

A further contribution of our study is the identification of prior augmentation as a risk factor for implant-related complications and re-operation. This was hypothesized previously by Spear in 2003 in the context of augmentation mastopexy and both compromised vascularity and increased biofilm burden are proposed etiologies.^27^ Four of the nine cases of capsular contracture occurred in patients with previous implants and the re-operation rate in those with previous augmentation or mastopexy was 31.1%. These observational findings support more cautious surgical planning in revisional cases. For example, when the direction of a previous pedicle is unknown, at least 1 year interval is required before revisional surgery, and the risk of NAC compromise must be emphasized during consent.

There are several limitations of the current study. Firstly, as a retrospective study, the study is exposed to selection bias and confounding. The decision to proceed with single-stage augmentation-mastopexy, and the operative steps (e.g., implant type, volume, mastopexy pattern) are individualized and influenced by surgeon preference and evolving practice over time. Furthermore, despite being one of the largest cohorts in the literature with long-term follow up, the number of adverse events were relatively few which limited the number of factors included in the multivariate analysis. Lastly, as a study of single-stage augmentation, the analyses were limited to the identification of risk factors and uncertainty remains on the indications of a two-stage procedure and its outcomes.^34^

In conclusion, single-stage augmentation mastopexy can be performed safely in carefully selected patients and long-term complication and re-operation rates are low. Risk stratification based on modifiable and non-modifiable patient factors is essential, and the use of a risk stratification tool may guide patient counseling and shared decision making. This study reinforces the growing recognition that, when offered with correct selection, the single-stage approach offers a predictable and efficient solution for patients with hypoplastic ptotic breasts.

## Funding

None.

## Declaration of competing interest

None declared.

## Appendix Supplementary materials

Image, application 1

Image, application 2
