# Supplementary figures and images for: Long-term outcomes after single-stage augmentation mastopexy: A ten-year series and risk stratification model
Source: JPRAS Open. 2026 Feb 28;49:272–83. doi: 10.1016/j.jpra.2026.02.026 (PMC13019983; doi:10.1016/j.jpra.2026.02.026)

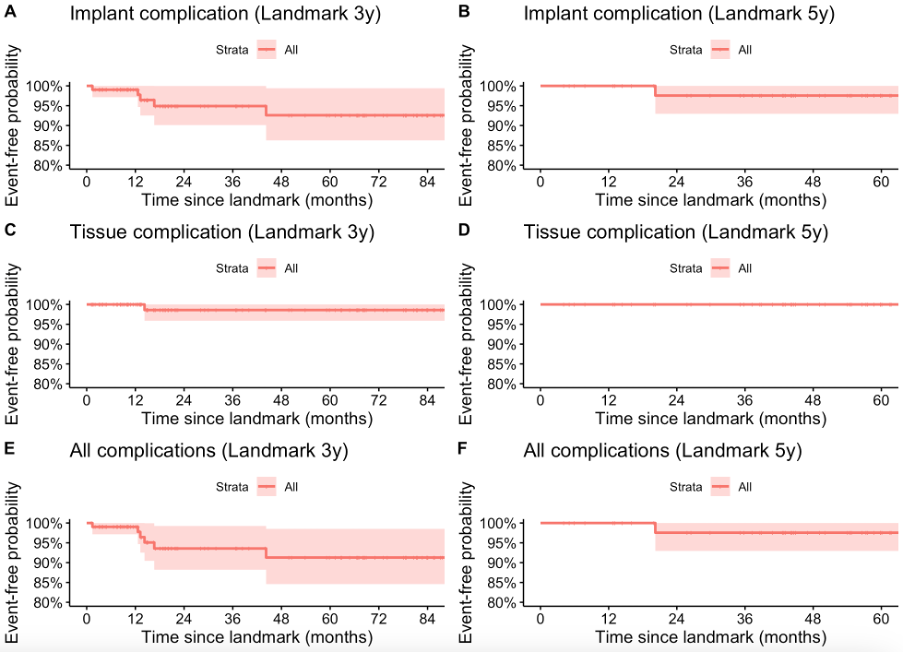

Supplement: Supplementary file 1 [file mmc1.zip › SupplementaryFigure1(revised)_V2.png]
