# Supplementary material for: Long-term outcomes after single-stage augmentation mastopexy: A ten-year series and risk stratification model
Source: JPRAS Open. 2026 Feb 28;49:272–83. doi: 10.1016/j.jpra.2026.02.026 (PMC13019983; doi:10.1016/j.jpra.2026.02.026)
Supplement: Supplementary file 2 [file mmc2.docx]

Supplementary Methods

Surgical techniques vary for augmentation mastopexy. However, the senior author (TS), who performed over two thirds of the cases in the unit, adopts the following approach. The breast meridian, the inframammary fold, the mid-line of the chest and edges of the sternum are marked (Supplementary figure 1a). The breast meridian is then extended below the inframammary fold onto the chest wall. Pitanguy’s point is then marked and estimates for the new upper margin of the areola are made according to measurement of the position of the new upper areola margin, the new diameter of the areola and Pitanguy’s point.^25^ The circumference of the new nipple areola is marked with a ‘mosque dome’ shape (14-15cm) and the circumference of the new NAC is then marked around the areola. The vertical limbs are marked by estimating the amount of pinch that can be removed in the lower pole of the breast, and the distance between vertical limbs on each side are compared and adjusted accordingly. If the vertical limbs are longer than 5-7cm, depending on the intended breast height or patients body habitus, horizontal excisions are required. In such cases triangular lateral lines are drawn to mark the horizontal closure line.

The patient, under general anaesthetic, is positioned supine and is prepped and draped. Overall, the 14-step plan described by Adams et al. is approximately followed.^26^ Markings are re-drawn, and a tourniquet is placed around the base of the breast. The initial incision around the area to be de-epithelialised is first performed and the skin de-epithelialised according to the proposed pedicle (Supplementary figure 1b). The planned skin excision is performed in the subcutaneous plane and the pedicle is isolated. If the upper margin of the old areola markings lie cranial to the lower margin of the new areola, then the NAC pedicle is planned with a superior vascular pedicle. The NAC flap is then raised. In superior NAC flap cases, the NAC is raised with a thickness of approximately 1cm to allow folding in of the NAC flap without compromising venous return (Supplementary figure 1c).

A vertical incision is made in the parenchyma in the midline of the breast, and lateral and medial breast flaps are raised. The flaps are subsequently rotated into the breast implant pocket. The breast implant pocket is then elevated and the implant is inserted (Supplementary figure 1d). When the pinch-thickness is less than 2cm a dual plane position is used, otherwise a subglandular pocket was most often used. To create an adequate pocket in the subglandular plane for the implant and to ensure sufficient mobilisation of the medial and lateral pillars, each pillar is mobilised (Supplementary figure 1e). Local anaesthetic and gentamicin are instilled to the implant pocket and implant, soaked in betadine, is introduced (Supplementary figure 1f). The edges of the wounds are undercut minimally with diathermy to ensure adequate folding in of each pillar. The implant is then inserted and vicryl sutures are used to suture the pillars and then skin.

Follow up was typically performed weekly until healing at a dressings clinic, at six weeks and six months by the operating surgeon, and beyond this time for patients with complications, as required.

Surgical steps for single-stage augmentation mastopexy: Patient marking (A); De-epithelialisation (B) Raising the nipple areolar complex flap (C); Creating the subglandular pocket (D); Mobilisation of the medial and lateral pillars (E); Implant insertion (F).


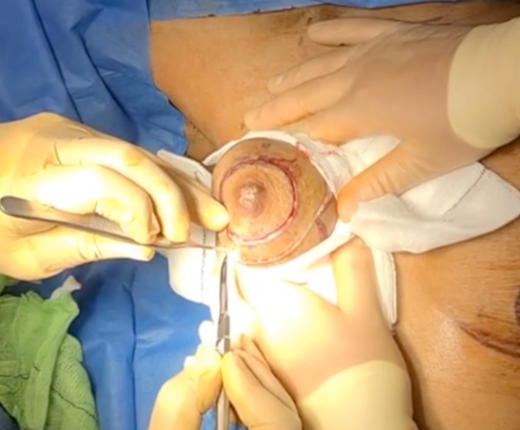

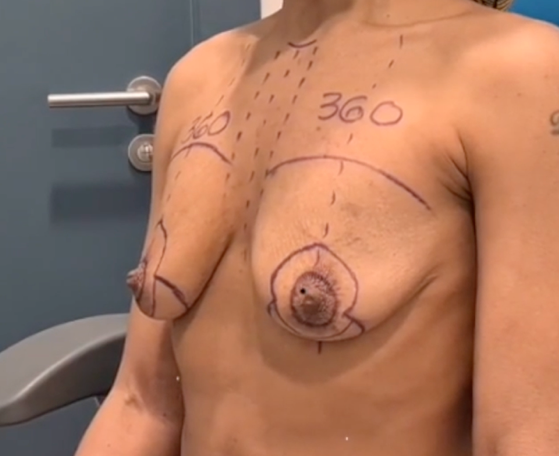


**A)**

**B)**


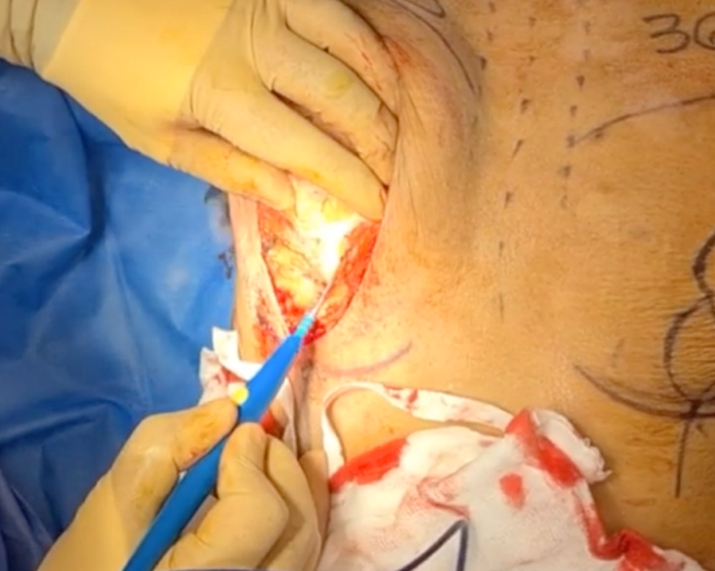

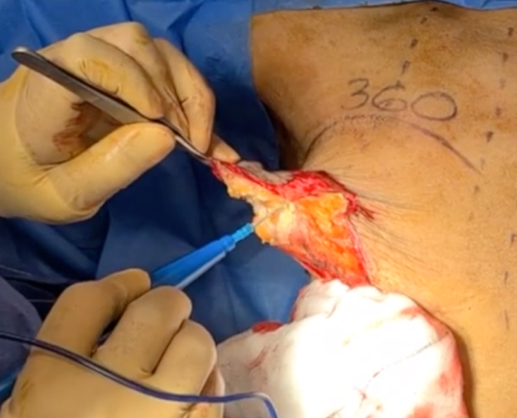


**C)**

**D)**


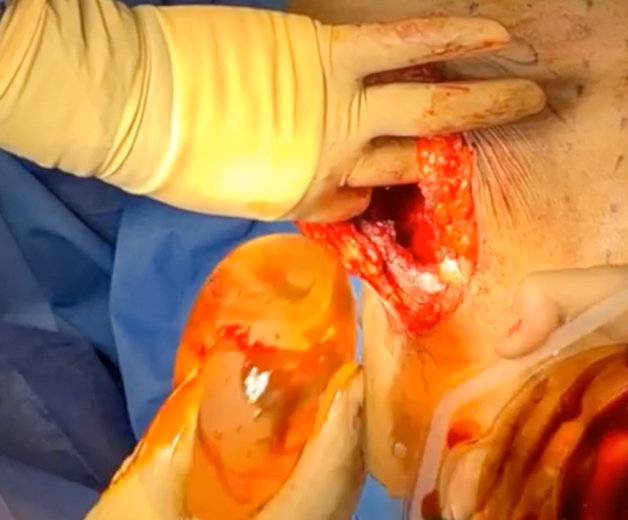
**
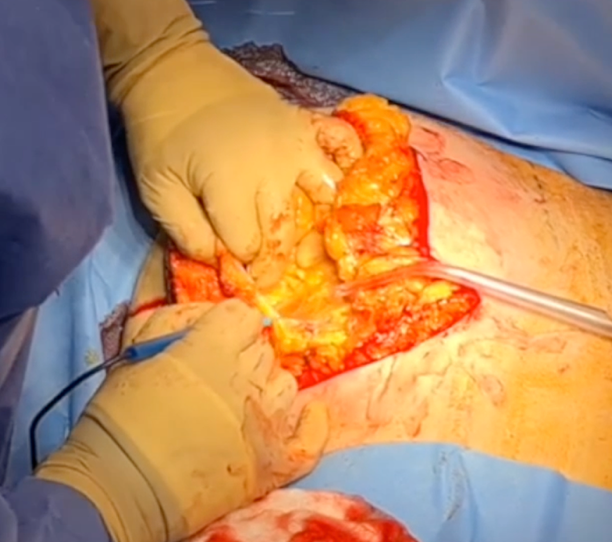
**

**E)**

**F)**
